# Maternal-fetal unit interactions and eutherian neocortical development and evolution

**DOI:** 10.3389/fnana.2013.00022

**Published:** 2013-07-19

**Authors:** Juan F. Montiel, Heidy Kaune, Manuel Maliqueo

**Affiliations:** ^1^Centre for Biomedical Research, Facultad de Medicina, Universidad Diego PortalesSantiago, Chile.; ^2^Nuffield Department of Obstetrics and Gynaecology, University of OxfordOxford, UK.; ^3^Laboratorio de Endocrinología y Metabolismo, Departamento de Medicina Occidente, Facultad de Medicina, Universidad de ChileSantiago, Chile.

**Keywords:** cerebral cortex development, placenta, maternal–fetal unit, evolution, serotonin, eutherians, transcriptome

## Abstract

The conserved brain design that primates inherited from early mammals differs from the variable adult brain size and species-specific brain dominances observed across mammals. This variability relies on the emergence of specialized cerebral cortical regions and sub-compartments, triggering an increase in brain size, areal interconnectivity and histological complexity that ultimately lies on the activation of developmental programs. Structural placental features are not well correlated with brain enlargement; however, several endocrine pathways could be tuned with the activation of neuronal progenitors in the proliferative neocortical compartments. In this article, we reviewed some mechanisms of eutherians maternal–fetal unit interactions associated with brain development and evolution. We propose a hypothesis of brain evolution where proliferative compartments in primates become activated by “non-classical” endocrine placental signals participating in different steps of corticogenesis. Changes in the inner placental structure, along with placenta endocrine stimuli over the cortical proliferative activity would allow mammalian brain enlargement with a concomitant shorter gestation span, as an evolutionary strategy to escape from parent-offspring conflict.

## INTRODUCTION

Placenta and brain are not recent innovations in vertebrate phylogeny (Aboitiz and Montiel, 2007; Renfree et al., 2013). Placental species emerge in a wide variety of taxa, even among invertebrates and basal vertebrates, involving multiple cases of analogous convergence (Blackburn, 1992; Renfree et al., 2013). In turn, brain origin can be tracked before the emergence of vertebrates (Aboitiz and Montiel, 2007). Both structures exhibit an important level of anatomical diversification across vertebrates and inside mammalian evolution. Placenta structural diversification is associated with life-history, and functional, genomics, and environmental requirements (Lewitus and Soligo, 2011), whereas brain shape variability is related to different functional dominances, behavioral repertories, and cognitive capacities (Krubitzer, 2007). The acquisition of a large brain size in mammalian evolution (Rowe et al., 2011) is mainly explained by the activation of developmental programs that allow a radial and tangential laminar expansion of the cerebral cortical surface (Cheung et al., 2007, 2010; Aboitiz and Montiel, 2012; Molnár and Clowry, 2012). These events are correlated with the activation, and functional specialization of cortical proliferative compartments, named ventricular zone (VZ) and subventricular zone (SVZ; Kriegstein et al., 2006; Molnár et al., 2006; Cheung et al., 2007, 2010; Molnár, 2011; Aboitiz and Montiel, 2012). These proliferative compartments are susceptible to be regulated by locally, nearby, and distantly originated signals. In this regard, brain development is dependent on (1) local cues and *in situ* cell-autonomous-specification (Franco et al., 2012), (2) neighboring information from telencephalic-signaling centers and developing connections (Dehay et al., 2001; Shimogori et al., 2004; Medina and Abellán, 2009; Aboitiz, 2011; Aboitiz and Montiel, 2012), and (3) distant systemic interactions that coordinate intrauterine and environmental regulations with brain development. This kind of control has been less explored in evolutionary neurobiology since most hypotheses about brain origin and evolution are focused on the intrinsic developmental control (local and neighboring signals) and functional properties of the brain (Karten, 1969, 1997; Aboitiz et al., 2003; Aboitiz and Montiel, 2007). During brain development, some of these distantly generated molecules participate in proliferative induction, myelination, cell differentiation, migration, growing of neuronal projections, and signaling (Vitalis and Parnavelas, 2003). In addition, they provide information to the fetus about environmental and maternal conditions through the placenta. In this review, we describe some maternal–fetal interactions and their plausible associations with brain evolution and development. In an attempt to integrate evolutionary, developmental, and genomic data, we discuss (1) the evolutionary origin of mammals, (2) the comparative morphology of placenta, (3) neocortical development, (4) placenta–brain endocrine interactions, (5) potential molecular placenta–brain interactions extracted from transcriptome databases, and (6) finally we speculate about a hypothesis for the neocortical expansion observed in mammalian evolution that integrates *in situ* and neighboring cues with the control based on endocrine signals from placenta, from which some represent new pathways that should be explored further.

## EVOLUTIONARY ORIGIN OF MAMMALS

The mammalian lineage arises from synapsids, a mammal-like reptile ancestor, diverged from other tetrapods about 300 million years ago (Mya) in the Carboniferous (geological period extended from ~359 to ~299 Mya; Gauthier et al., 1988). The early mammal-like reptile Probainognathus had slender and elongated cerebral hemispheres bearing a small dorsal slope that is believed to be a forerunner of the neocortex (Quiroga, 1980; Aboitiz et al., 2003; Aboitiz and Montiel, 2007). Synapsids gave rise to pelycosaurs, lizard-like animals succeeded in the late Permian (extended from ~299 to 252 Mya) by the therapsids (Kemp, 2006). Most therapsids became extinct by the end of the Triassic period (extended from ~252 to ~201 Mya), but one group of carnivorous therapsids, called cynodonts, survived well into the Jurassic period (extended from ~201 to ~145 Mya; Carroll, 1988). Early cynodonts had a restricted sensory repertory and a poor sensory–motor integration with a relatively low encephalization quotient (EQ; a measure of relative brain size as a function of the total body size; Rowe et al., 2011). From cynodonts arose the eucynodonts or mammaliaforms, this group includes Jurassic fossils such as Sinoconodon and Morganucodon, whose gross morphology resembled that of some present-day insectivores (currently known as order Eulipotyphla; Rowe, 1996; Kaas, 2013). Eucynodonts differ from their predecessors by having an increased olfactory sensitivity, improved tactile resolution, and motor coordination (Rowe et al., 2011), which are functional changes that would contribute primarily to a first pulse of pre-mammalian encephalization (Rowe et al., 2011). This hemispheric expansion differentiates mammaliaforms from mammal-like reptiles and most other vertebrates (Rowe et al., 2011). Considering this evidence, it has been proposed that the brain expansion was a late event in the lineage leading to mammals, more or less coincident with the acquisition of modern mammalian characters observed in fossils like the basal mammaliaform, Morganucodon, or in the closest known extinct to mammals, Hadrocodium (Kielan-Jaworowska et al., 2004; Aboitiz and Montiel, 2007; Rowe et al., 2011). Mammalia arose from eucynodonts in/or before the Early Jurassic (~200 Mya; Kielan-Jaworowska et al., 2004). These ancestral mammals were characterized by an expansion of the olfactory sensory system, which has been linked to a genomic amplification of the olfactory receptors (Niimura, 2009; Rowe et al., 2011). In some descendant clades, the olfactory system was further elaborated, whereas in others it was reduced and replaced by other sensory modalities (Aboitiz et al., 2003; Aboitiz and Montiel, 2007; Krubitzer, 2007; Rowe et al., 2011). Only much later, acute visual and auditory systems evolved among mammals (Luo et al., 2011; Aboitiz and Montiel, 2012).

**FIGURE 1:**
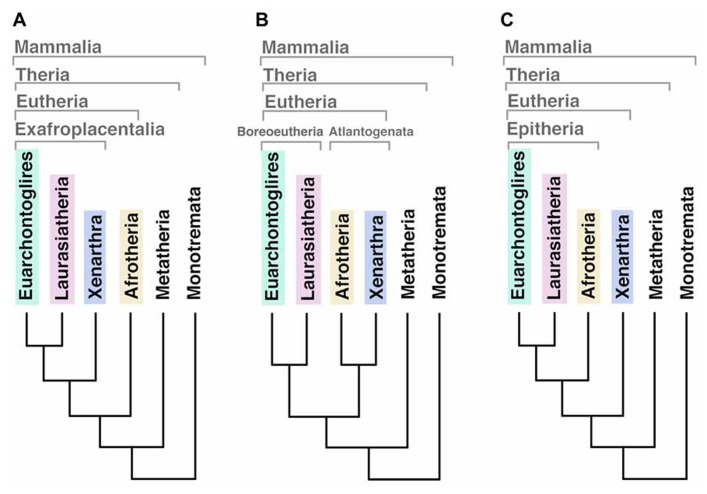
**Different outputs for the basal relationships among eutherian mammal superordinal clades.** **(A)** Afrotheria as the most basal mammalian clade, sister lineage of exafroplacentalia (all other eutherians, also called notolegia). **(B)** Atlantogenata (Afrotheria and Xenarthra) as sister lineage of Boreoeutheria (Euarchontoglires and Laurasiatheria). **(C)** Xenarthra as the most basal clade, sister lineage of Epitheria (all other eutherians).

Three mammalian subclasses became extinct in different evolutionary moments. Triconodonta, the earliest lineage diverged in mammalian phylogeny, disappeared at the end of the Cretaceous period (extended from ~145 to ~66 Mya) leading to a lack of basal-related in living mammals. The second was the Multituberculata, and the latest extinguished was the infraclass of Theria called Palaeoryctoides. A recent analysis suggests that the first modern placental orders (eutherians) emerged around 2–3 million years later than the Cretaceous–Paleogene (K–Pg) extinction event occurred 66 Mya (O’Leary et al., 2013). It was predicted that these ancestors had a hemochorial placenta with trophoblast, gyrencephalic cerebral cortex and relatively high EQ (over 0.25) when compared with other vertebrates (O’Leary et al., 2013). Phylogenetic analyzes of the living eutherian mammals identify four primary superordinal clades: Afrotheria, Xenarthra, Euarchontoglires, and Laurasiatheria (for a list of species belonging each group please see **Table 1**). The basal diversification of eutherians has been historically considered an unstable node (O’Leary et al., 2013) and the phylogenetic relationship between these lineages is still under debate since different analyzes have generated different outputs of ancestry (**Figure 1**). Using 18 homologous genes segments, Afrotheria was originally positioned as the most basal mammalian clade, with Xenarthra as the second, and Euarchontoglires and Laurasiatheria as the third branches of the mammalian tree (Murphy et al., 2001; **Figure 1A**). Using genomic sequences, the phylogenetic relationship of mammals has been re-informed (Murphy et al., 2007) and “confirmed” (Prasad et al., 2008), positioning Atlantogenata (Afrotheria and Xenarthra) together as sister lineages of Boreoeutheria (Euarchontoglires and Laurasiatheria; **Figure 1B**). A recent publication renewed this debate resolving this basal relation as a split between Xenarthra and Epitheria (Afrotheria, Laurasiatheria and Euarchontoglires; O’Leary et al., 2013; **Figure 1C**).

**Table 1:** Primary clades of eutherian living mammals.

Class	Supercohort	Infraclass	Superorder	ORDER and/or Suborder; animal examples/
Mammalia	Theria	Eutheria	Afrotheria	AFROSORICIDA: Chrysochloridea; golden mole/ Tenrecidae; tenrecs/ MACROSCELIDEA; elephant shrews (sengis)/ TUBULIDENTATA; aardvarks/ HYRACOIDEA (hyraxes); rock hyrax/ PROBOSCIDEA; elephants/ SIRENIA; sea cows (dunging and manatees)
			Xenarthra	Vermilingua; anteaters/ Folivora; tree sloths/ CINGULATA; armadillos
			Euarchontoglires	RODENTIA; rat, mouse, capybara/ LAGOMORPHA; rabbits and hares, treeshrews/ DERMOPTERA; colugos/ PRIMATES; prosimians and simians
			Laurasiatheria	EULIPOTYPHLA; shrews, hedgehogs/ PHOLIDOTA, pangolins/ CHIROPTERA; bats/ CETACEA; whales/ ARTIODACTYLA; most hoofed mammals (such as hippopotamuses)/ CARNIVORA; cats, dogs, bears, seals, etc
		Metatheria		DIPROTODONTIA; kangaroo, koala, possum, wombat/ DASYUROMORPHIA; tasmanian devil, quolls, dunnarts, numbat/ MICROBIOTHERIA; monito del monte/ PERAMELEMORPHIA; bilbies and bandicoots/ NOTORYCTEMORPHIA; Marsupial moles/ DIDELPHIMORPHIA; opossums/ PAUCITUBERCULATA; shrew opossums.
	Prototheria			MONOTREMATA; Platybus, echidnas

## COMPARATIVE MORPHOLOGY OF PLACENTA

Understanding the organization of the phylogenetic tree of mammals allows the visualization of the evolutionary history of different traits and the definition of the presumed ancestral structure of the placenta (Wildman et al., 2006). The acquisition of the placenta implies as first requirement, the emergence of viviparity (live-bearing) since this made possible the elaboration of specialized structures that allowed the development of the eggs within the maternal body, providing nutrition and protection. It has been postulated that placenta has evolved concomitantly with the viviparity more than 100 times in different lineages of non-mammal amniotes (Crespi and Semeniuk, 2004). The most ancient evidence of viviparity in amniotes arise from fossils of mosasauroids, a cretaceous marine lizard, containing embryos along the posterior trunk region (Caldwell and Lee, 2001). The reptile ancestor of mammalian lineage was oviparous (egg-laying) since in reptiles viviparity has evolved more recently than in mammals (Blackburn, 1992). The mammalian common ancestor of monotremes (which lay eggs) and therians was presumably egg-laying as well (Oftedal, 2002). Supporting evidence for this is that in reptiles oviparity can evolve into viviparity via a sequential increase in the duration of egg-retention, as it has been seen in lizards and snakes (Blackburn, 1992). Once committed to viviparity, the eggshell membrane thickness is drastically reduced, thus, return to oviparity from viviparity has not been seen in amniotes (Oftedal, 2002).

**FIGURE 2:**
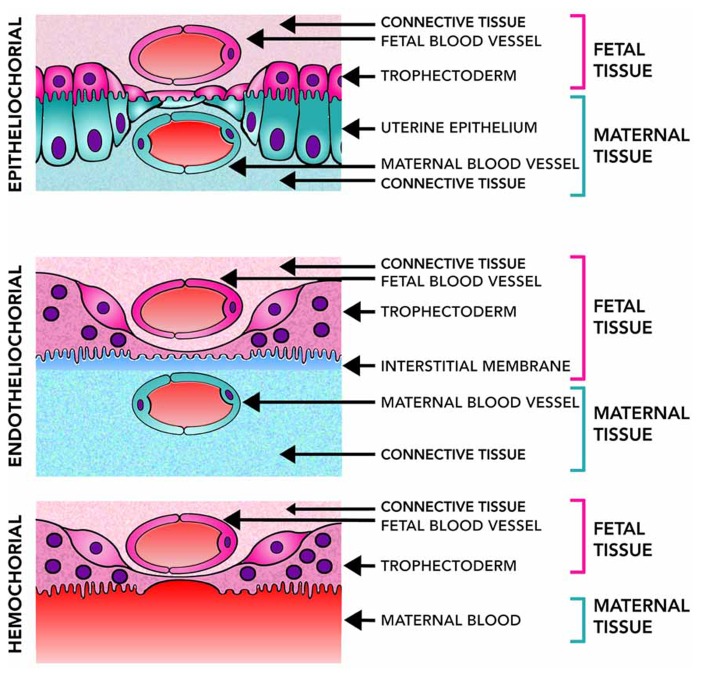
**Placental level of invasiveness.** The placenta varies across mammalian species in the invasiveness and their access to maternal blood flow. Epitheliochorial placentas, the least invasive, have three layers of maternal tissue separating the fetus from maternal blood. Endotheliochorial placentas are partially invasive and only the endothelial wall of the maternal blood vessels, and some interstitial tissue, separates the fetus from the maternal blood. Hemochorial placentation is the most invasive and allows fetal tissues to be bathed directly in the maternal blood. Epitheliochorial and Endotheliochorial placentas can be found in Afrotheria, Euarchontoglires and more frequently in Laurasiatheria. Hemochorial placentas are found in all eutherian superorders (Wildman et al., 2006), showing a broad distribution of placental types in the mammalian class.

The diversity of placental structures found among species is remarkable. Differences in placental shape, degree of the relationship between the chorion and uterine wall, number of layers of trophoblast, shape of maternal–fetal interdigitation (villous, trabecular or labyrinthine), variations in the interhemal barrier mainly characterized by different degrees of hypertrophy of maternal endothelium and presence of cytotrophoblast and/or syncytial trophoblast are commonly observed (Enders and Blankenship, 1999; Enders and Carter, 2004). Three main types of placentas can be recognized according to the extent of how the fetal tissue invades the wall of the uterus or the maternal vessels. In general, epitheliochorial placenta is an extensive and diffuse structure, lining the uterine wall, and exhibiting limited invasiveness without trophoblast invasion of uterine vessels. In the endotheliochorial placenta, a network of maternal capillary grows within the trophoblast, allowing a better exchange between the mother and fetus and reducing the risk of passing fetal cells into the maternal circulatory system. Finally, in the hemochorial placenta, the maternal blood is in direct contact with the trophoblast, which has the advantage of a more efficient nutrient uptake and waste elimination (**Figure 2**). This extensive maternal–fetal communication also implies some disadvantages like a major risk of maternal bleeding after delivery and a greater chance of fetal cells transfer to the maternal system (Enders and Carter, 2004).

## MORPHOLOGICAL PLACENTAL ORGANIZATION ACROSS LIVING MAMMALIAN SPECIES IS ASSOCIATED WITH SHORTER GESTATIONS AND MATERNAL INVESTMENT SPAN REDUCTION

The eutherian lineage displays a huge placentation diversification. Mess and Carter (2006) developed an exhaustive analysis of 19 morphological features and degrees of development at birth across 35 mammalian species, getting a plausible placental profile of the stem ancestor of living mammals (Carter and Mess, 2007; Capellini, 2012). The authors used a cladistics analysis placing Afrotheria and Xenarthra as sister to other eutherians, which agrees with the phylogeny of living mammals obtained from genomic data (Murphy et al., 2007; Prasad et al., 2008). Together with recent studies focused on defining the ancestral structure of the eutherian placenta, these studies agreed that this ancestral placenta had a hemochorial interface, discoid shape, and labyrinthine maternal–fetal interdigitations (Wildman et al., 2006; Capellini, 2012). Although the placenta organization is highly variable between mammals, the ancestral hemochorial and discoid placenta structure has been preserved in haplorhine (tarsiers, new and old word monkeys, and apes) primates (Vogel, 2005; Wildman et al., 2006; Lewitus and Soligo, 2011). As these big-brained mammals conserve the ancestral placental organization and share this feature with small-brained mammals it is questionable that structural differences in placenta can account for brain size expansion across mammalian evolution. However, different structural placental features have been associated with brain enlargement, leading to conflictive conclusions. One of these relies on the brain as a highly expensive organ to grow and maintain (Elliot and Crespi, 2008), so it was proposed that a highly invasive hemochorial placentation (**Figure 2**) is necessary for fetal brain growth. However, this relation is not supported once the analyzes are refined (Sacher and Staffeldt, 1974; Capellini et al., 2011). Even more, dolphins, as humans, have a relative larger brain; nevertheless, they possess an epitheliochorial placenta (**Figure 2**). Accordingly, invasiveness of the placenta is not a requirement to develop large brains (Martin, 2003).

Bias in the inclusion of relatively small-brained marsupials against the largest-brained placental mammals (Laurasiatheria and especially Euarchontoglires), or the addition of preimplantation stages lacking placenta formation would hamper these comparisons between marsupial and eutherian mammals (Weisbecker and Goswami, 2010; Capellini et al., 2011). Recently, Capellini (2012) analyzed different placental attributes, concluding that whereas invasiveness association to fetal and brain growing is not supported by comparative studies, species with highly interdigitated labyrinthine placentas produce neonates of similar body and brain size but in less than half the gestational time than those associated with less interdigitated (villous and trabecular) placentas. Capellini suggested that the effects of placental interdigitation on growth rates and the way that these are traded off against gestation length may be important for understanding the evolutionary dynamics of parent-offspring conflict.

## NEOCORTICAL DEVELOPMENT

Most neurons in the neocortex derive from multipotent neural stem cells in the proliferative epithelium of the VZ lining the ventricular surface of the telencephalic wall. In the VZ, radial glial cells will generate lower- and upper-layer neurons according to distinct fate potentials (Franco et al., 2012), the Cux2 negative radial glia first produces excitatory neurons, most of which migrate radially to make up the embryonic preplate and the deepest cortical layers, instead Cux2 positive radial glia are fated to generate upper-layer neurons (Franco et al., 2012). Later in development, divisions of the Cux2 positive radial glia produce cells called intermediate progenitors, that detach from the ventricular surface and aggregate in a zone overlying the VZ (Kriegstein et al., 2006; Franco et al., 2012), the SVZ, a second proliferative compartment that is under control of Pax6 transcriptional factor and express *Svet1*, *Cux2*, and *Tbr2* genes. In the SVZ, cells undergo one to three more cell divisions and then migrate to build up the superficial layers of the neocortex. Neurons generated in successively later moments are incorporated into progressively more superficial layers, generating the inside-out neurogenetic gradient that is characteristic of the neocortex.

## THE ACTIVATION OF PROLIFERATIVE COMPARTMENTS AS A STRATEGY FOR NEOCORTICAL EXPANSION

**FIGURE 3:**
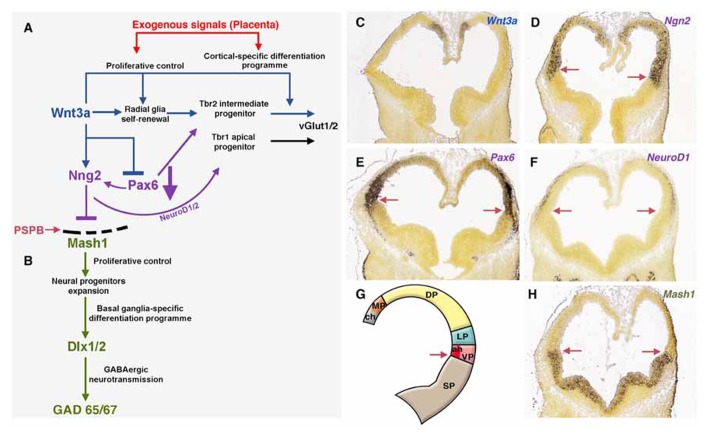
** Exogenous signals from placenta would complement telencephalic-signaling control during brain development.** **(A)** The upregulation of Wnt3a trough the canonical Wnt pathway induce self-renewal of radial glia, early differentiation of Tbr2 cortical intermediate progenitors (Munji et al., 2011) and restrict the expansion of the ventral pallium (antihem) driven by Pax6. The antihem express secreted Frizzled-Related Proteins (Sfpr1 and 2, not shown) that neutralize the action of dorsally derived signals like Wnts. Pax6 activates the expression of the dorsal proneural factor neurogenin 1/2 with the consequent activation of NeuroD1/2 and inhibition of Mash1, a proneural factor highly expressed in subpallial domains. Thus, the pallial/subpallial boundary is defined by the limit of expression of Ngn2 and Mash1 (red arrow in **A**, **D–H**). **(B)** Mash1 induces a first group of transcripts in the phase of expansion of neural progenitors (Castro et al., 2011) and activates a second group of genes at the subsequent phases of cell cycle exit and neuronal differentiation (Bertrand et al., 2002). **(C–F)** *In situ* hybridization (ISH) expression pattern of signaling factors in mouse at developmental stage E11.5 **(G)** Gene expression of signals/factors that helps to define the telencephalic compartmentalization. **(H)** Subpallial expression of *Mash1* at E11.5. All ISH images were taken from the Allen Developing Mouse Brain Atlas, available from: . ch, cortical hem; LP, lateral pallium; MP, medial pallium; VP, ventral pallium; SP, subpallium.

According to recent models of neocortical growth, early tangential expansion of the neocortex is based primarily on the divisions of primary progenitors, which enlarge the surface of the VZ, and later on the tangential growth and radial thickening (generation of superficial layers) of the neocortex depending mainly on the proliferation of cortical intermediate progenitors (Dehay and Kennedy, 2007; Pontious et al., 2008) and glial-like neurons located in the SVZ (Reillo et al., 2011; Wang et al., 2011b; Molnár et al., 2011). From a comparative perspective, there seem to be an increased cell number in mammals in an arbitrary unit column of cortex (Cheung et al., 2007, 2010). Adult mice or macaques possess a significantly higher number of cerebral cortical neurons compared with marsupials (Cheung et al., 2007, 2010). The presence of intermediate (or basal) progenitor cell divisions and gene expression patterns suggest that the SVZ emerged prior to the Eutherian–Metatherian split and it might have been the major driving force behind the evolution of the six-layered neocortex in mammals (Cheung et al., 2007, 2010; Aboitiz, 2011; Aboitiz and Montiel, 2012; Molnár and Clowry, 2012). Interestingly, while a VZ has been described in all vertebrates that have been studied, a distinctive dorsal pallial SVZ appears only in some species. Among mammals, the SVZ extends from the lateroventral aspect of the hemisphere to the dorsal pallium. Across species, the growth of the SVZ appears to correlate with the development of the superficial neocortical layers, being especially complex in primates and minimal in marsupials (Cheung et al., 2010; Molnár, 2011). Underlying the neurogenetic development, there is a molecular regionalization process in which the cortical neuroepithelium acquires its identity on the basis of the expression of regulatory genes that control the pattern of differentiation, yielding its characteristic adult phenotype. Molecular evidence indicates that the embryonic cerebral hemispheres are patterned according to several signaling centers from which morphogens are produced and expressed in gradients in different directions (Sur and Rubenstein, 2005; O’Leary and Sahara, 2008; Medina and Abellán, 2009). Thus, modulation of such gradients may yield to important changes in brain development, expanding some regions and reducing others (Medina and Abellán, 2009; Aboitiz, 2011; Aboitiz and Montiel, 2012; **Figure 3**). We believe that several traits of cortical neurodevelopment would account for the high mean EQ of Euarchontoglires and especially of large-brained primates (Aboitiz and Montiel, 2012; Molnár and Clowry, 2012). Primates developed a subcompartmentalized neocortical proliferative SVZ (García-Moreno et al., 2012), and an extra-laminated transient subplate (Wang et al., 2010, 2011a; Montiel et al., 2011). Together with other changes, these characteristics allowed a radial and tangential cortical expansion and consequently allowed to alter the conserved brain design that primates inherited from early mammals (Aboitiz and Montiel, 2012).

## PLACENTA–BRAIN ENDOCRINE INTERACTIONS

In some way, placenta resembles the function of several endocrine systems; thus it is positioned as the main endocrine organ throughout intrauterine development. Placenta produces and releases a number of signaling substances including cytokines, neuropeptides, neurosteroids, and amines (Petraglia et al., 1996). Some of them are known for influencing fetal brain development, e.g., regulating the synthesis of neuroactive factors and corticogenesis (Petraglia et al., 2010). Placenta is a crucial regulator of maternal–fetal interactions (Hsiao and Patterson, 2012). Indeed, structural changes in placenta are associated with the development of diseases in later life (Barker et al., 1990). Moreover, placenta’s roles in regulating nutrient transport, endocrine function and immune tolerance are involved in growth restriction, hypoxia, and neurological complications (Fernandez-Twinn et al., 2003; Jansson and Powell, 2007). Several functional pathways associate this organ with brain development and, recently, reciprocal interactions from the brain to placenta have been proposed (Ugrumov, 2010). In addition, the tightening of the blood–brain barrier is a gradual process, with an earliest angiogenesis phase occurring during early brain developmental stages (E13–E14 in rat). This is characterized by a high paracellular permeability (Liebner et al., 2011) and therefore permitting fluent molecular interactions, which potentially allows the placenta to participate in brain development (Bonnin et al., 2011).

## SEROTONIN AND NEUROGENIC CONTROL

**FIGURE 4:**
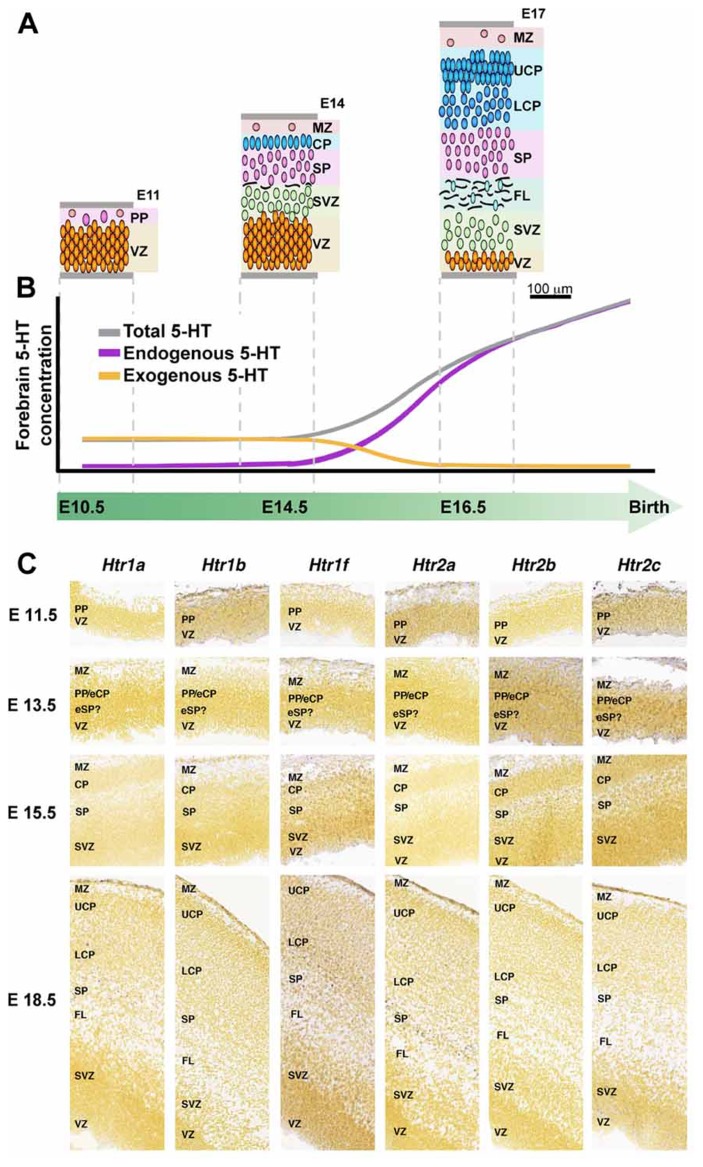
** 5-HT and 5-HT receptors in mouse developing cerebral cortex.** **(A)** Organization of the developing mouse cortex at E11, E14, and E17, modified from Smart et al. (2002) with permission. **(B)** Dynamic of 5-HT brain sources during intrauterine development, early exogenous (placenta; orange line) switch to a latter endogenous (5-HT brainstem axons, purple line) 5-HT source, modified from Bonnin et al. (2011), with permission. **(C)** Expression of 5-HT receptors during mouse cortical development, ISH obtained from Allen Developing Mouse Brain Atlas, available from: . CP, cortical plate; eCP, early cortical plate; eSP, early subplate; FL, fiber layer; LCP, lower cortical plate; MZ, marginal zone; PP, preplate; SP, subplate; SVZ, subventricular zone; VZ, ventricular zone; UCP, upper cortical plate.

One of these maternal–fetal interactions is through serotonin (5-HT) pathway. This neuroactive factor has been associated with proliferative activity, migration, and differentiation processes during neocortical development (Vitalis and Parnavelas, 2003). Originally, it was though that endogenous sources of 5-HT were responsible for the stimulation of corticogenesis, but since there is a mismatch between this endogenous generation of 5-HT (serotoninergic axons reach the corticostriatal junction at E16 in rats) and the peak of cortical neurogenesis (E12–E17; Vitalis and Parnavelas, 2003), placenta/brain interaction could not be explained by endogenous sources of 5-HT (**Figure 4**). Instead, the exogenous source of 5-HT produced by the placenta is required to maintain normal levels of forebrain 5-HT during early stages of brain development (Bonnin et al., 2011) and would explain the developmental-functional association between 5-HT and cortical development. Receptiveness to 5-HT during early stages of cerebral cortical development depends on the expression of 5-HT receptor subtypes in the developing cortex (Lidow and Rakic, 1995; Vitalis and Parnavelas, 2003). For an accurate expression mapping of 5-HT1 receptors in mouse see Bonnin et al. (2006). Interestingly, in monkeys, high levels of 5-HT receptors expression have been reported in the proliferative zones of the occipital lobe during neurogenesis (Lidow and Rakic, 1995). Moreover, some 5-HT receptors have been shown to be functional before birth, suggesting that they may orchestrate early steps of cortical development. A postnatal injection of 5-HT1 receptors agonist increases the number of hippocampal precursors and neurons (Vitalis and Parnavelas, 2003), instead hippocampal proliferation of precursors is repressed when the 5-HT synthesis is pharmacologically inhibited (Brezun and Daszuta, 1999; Vitalis and Parnavelas, 2003). 5-HT stimulation also promotes differentiation, and therefore it is not clear whether 5-HT stimulation mediates proliferation or simply speeds up the cell cycle (Vitalis and Parnavelas, 2003). Interestingly, embryonic pharmacological depletion of 5-HT (Vitalis and Parnavelas, 2003) and cocaine administration (which interacts with the 5-HT pathways; Clarke et al., 1996), both induce microcephaly. Elevated 5-HT output associated with a reduced 5-HT transporter and 5-HT1A receptor have been observed in hippocampus of adult animals that underwent prenatal stress during fetal life, supporting the role of prenatal imprinting on behavioral alterations in adult life (Van den Hove et al., 2006; Mueller and Bale, 2008).

## PLACENTAL STEROIDS AND BRAIN DEVELOPMENT

During pregnancy in mammals, placenta produces a large amount of steroids, which are crucial for the survival, development, and health of the developing embryo. Progesterone and estrogens (estrone, estradiol, estriol, and its conjugated forms) are the main hormones during pregnancy. Progesterone is fundamental to modulate the maternal immune response allowing the maternal tolerance of the fetal “semi-allograft.” On the other hand, estrogens are needed to promote the placental growth and angiogenesis and influence fetal growth and metabolism. These steroids are in extremely high concentrations in maternal circulation. However, placental tissue can metabolize them to inactive forms in order to avoid fetal exposure. Many of these metabolites can act as neurosteroids in adult brain, however, their contribution to fetal brain development remains under debate. For example, allopregnanolone, a progesterone metabolite modulates the activity of GABAergic and glutamatergic neurons in fetal brain and may mitigate the brain injury provoked by asphyxia in the hippocampal region, since allopregnanolone can control the proliferation and apoptotic patterns in cerebellum and hippocampus (Nicol et al., 1999; Yawno et al., 2007, 2009).

Similar to sex steroids, glucocorticoids levels are lower in fetal than in maternal circulation. This difference is attributable to the high expression of 11β hydroxysteroid dehydrogenase type 2 (11β-HSD2) in both the placenta and fetus. In the placenta, 11β-HSD2 catalyzes the rapid inactivation of cortisol and corticosterone to inert 11 keto-products, and then it serves as a “glucocorticoid barrier,” modulating the transfer of glucocorticoids to the fetus. In the placenta, 11β-HSD2 is highly expressed in the syncytiotrophoblast of humans and in the labyrinthine zone of rodents (Brown et al., 1996; Waddell et al., 2012). Interestingly, animal models of maternal stress have been associated with lower expression of placental 11β-HSD2 and low birth weight in rodents, suggesting a relationship with fetal programming (Fowden et al., 2008).

In general, normal concentrations of glucocorticoids are essential in the development of many organs, including central nervous system. However, some conditions associated with elevated levels of glucocorticoids, as stress or reduced capacity of placenta to metabolize it, can leads to detrimental effects on brain development and long-term behavioral effects, since glucocorticoid receptors are highly expressed in some brain areas like hippocampus (Reul and de Kloet, 1985). Primate models of stress and dexamethasone-exposure during pregnancy, exhibit degenerative changes and reduction of brain volume, associated with lower number of neurons in the hippocampus (Uno et al., 1989, 1990; Coe et al., 2003). Moreover, these effects are maintained at least until 2 years after birth, suggesting a possible long-term effect in learning process and memory (Uno et al., 1994). In cerebral cortex, studies conducted in rodents have demonstrated a reduction in dendritic arborization and synaptic loss in frontal cortex of males but no effects were observed in females indicating a gender-specific mechanism of action (Barros et al., 2006). In addition, cerebellum exhibits a reduction in the volume fraction of granule cells nuclei in the granular layer with less synaptic density (Ulupinar and Yucel, 2005). Some evidence points out that prenatal glucocorticoid exposure can impact on serotonergic and catecholamine pathways. Therefore, we can hypothesize that the mechanism associated with endocrine function in placenta can impact brain development, probably playing a central role on brain evolution. However, the information about comparative endocrine function in different species is still limited, making it difficult to interpolate these functions to the eutherian ancestor.

## EXPLORING POTENTIAL MOLECULAR PLACENTA–BRAIN INTERACTIONS FROM TRANSCRIPTOME DATABASES

**FIGURE 5:**
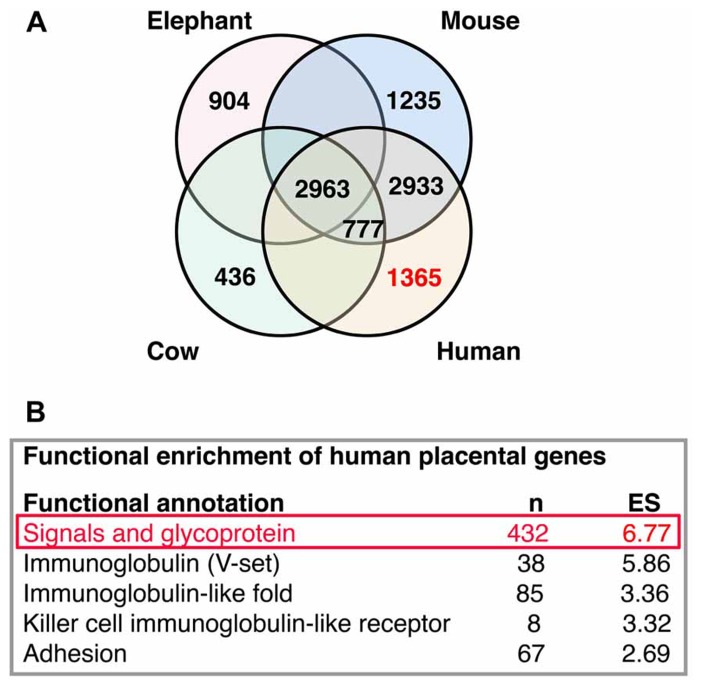
** Interactions analysis.** **(A)** Distribution of placental transcripts obtained by Hou et al. (2012) and reproduced with permission. **(B)** Top five functional enrichments of human species-specific placental transcripts obtained by Hou et al. (2012) and reproduced with permission. The top one enriched module (in red) was further analyzed in **Figure 6**.

High-throughput analysis of active transcripts is a powerful tool to explore possible molecular interactions across organs and species. Brain and placenta share some remarkable transcriptional features as the expression of imprinted genes and the expression of transcriptional pathways of immune interactions. Transcriptomes from placenta (Hou et al., 2012; **Figure 5A**) and developing neocortical brain compartments (Ayoub et al., 2011) can be clearly differentiated between different mammalian species (Ayoub et al., 2011; Hou et al., 2012). Comparing placenta transcriptomes of representative species from three eutherian superorders: elephant (Afrotheria), cow (Laurasiatheria), mouse, and human (Euarchontoglires), Hou and collaborators found 2,963 genes commonly expressed and a variable number of active transcripts with species-specific expression (elephant, 904; cow, 436; mouse, 1,235; and human, 1,365; Hou et al., 2012; **Figure 5A**). At this point of our on-going studies, we become interested in the published list of human placental differentially expressed genes (Hou et al., 2012), this species-specific set of genes exhibit significant functional enrichment (the top five are reproduced from originals in **Figure 5B**). The top one gene module is enriched in signal molecules (this module was originally labeled as glycoproteins, but we renamed it after reproduce this analysis to represent its enrichment in signal genes, **Figure 5B**).

**FIGURE 6:**
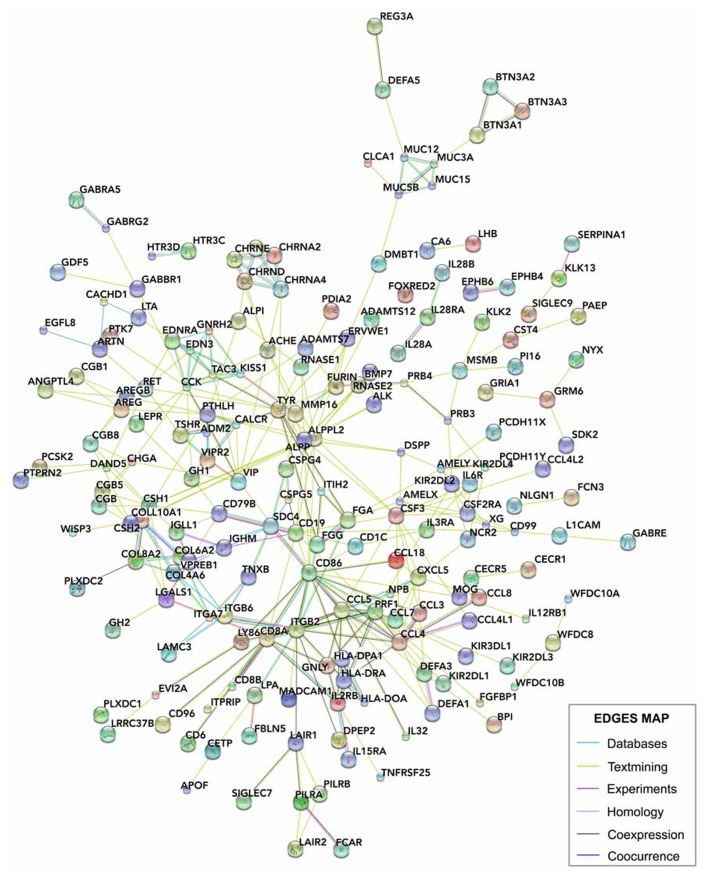
** STRING analysis of the interactions between placental genes.** The signals and glycoprotein enriched module of human placental transcripts (**Figure 5B**) was analyzed using STRING 9.05 (), a database of known and predicted protein interactions, which responds by displaying a network of nodes (genes) connected by colored edges representing functional relationships. Interactions of these genes were identified based on the evidence indicated in the edges map. Unconnected genes were removed.

We compared these human placental signal-enriched transcripts, and those transcripts from human developing cortex (including the VZ, inner and outer SVZs, and cortical plate; Fietz et al., 2012) looking for systemic protein–protein and general functional interactions. To do this, we investigated known and predicted protein interactions based on genomics, coexpression, and literature data using STRING 9.05 (), a network analysis database focused on protein interactions (Szklarczyk et al., 2011; Franceschini et al., 2013; Lv et al., 2013; **Figure 6**).

**FIGURE 7:**
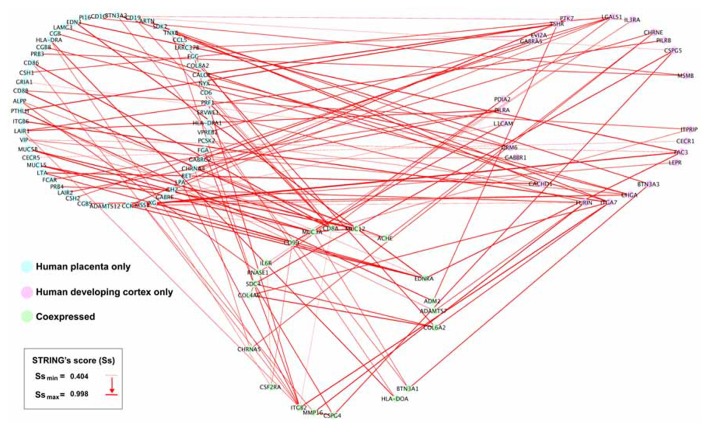
** Placental interactions with the developing human cerebral cortex.** From the subset of interactions generated from the STRING analysis (**Figure 6**), we manually selected those genes interacting with human developing cerebral cortex genes. Interacting human placental, human developing cerebral cortex genes, and pairwise STRING’s interaction scores were imported into Cytoscape 2.8.2. The human placenta–brain network was constructed using a JGraph circle layout. Using NetworkAnalyzer, the interactions across nodes were visualized in red lines, and the thickness of the edges was weighted using the STRING’s score (Ss), which indicates the level of interaction between nodes.

We obtained 341 potential interactions from human placenta active transcripts, ranging a STRING’s score from 0.404 to 0.998, and initially not restricted to any target tissue. Then, we manually projected this list onto the developing human cortex transcriptome (Fietz et al., 2012) in order to detect plausible gene placenta–brain interactions, obtaining 112 interactions, involving 73 transcripts expressed only in the human placenta 20 transcripts coexpressed in the human placenta and the human developing cortex, and 24 transcripts expressed only in the human developing cortex. The genes expressed in the developing cortex and their placenta interacting counterparts have been represented as a gene network of protein interactions in the **Figure 7**.

## PLAUSIBLE PLACENTA–BRAIN INTERACTIONS PREDICTED BY TRANSCRIPTOMIC AND PATHWAY ANALYZES

Some of the identified pathways have been associated directly or indirectly to proliferative activation, cell migration, and differentiation during cortical development, and related to several neurological disorders. *CSPG5* (chondroitin sulfate proteoglycan 5) is expressed predominantly in the developing cortex participating in dendrite branching and synapses formation (Aono et al., 2000). CSPG5 interacts with the cell surface protein CD19 expressed in the placenta, with the *CSPG4* proteoglycan (chondroitin sulfate proteoglycan 4) and with the transmembrane protein *SDC4* (syndecan 4) placenta–brain coexpressed genes. Interestingly, transcriptomic analyzes have suggested that cell adhesion and cell–extracellular matrix interactions promote the proliferation and self-renewal of neural progenitors in the developing human neocortex (Fietz et al., 2012). *LGALS1* (lectin, galactoside-binding, soluble, 1), expressed in the developing cortex, encodes for galectin, a known regulator of neural stem cell proliferation (Sakaguchi et al., 2006; Imamura et al., 2008) and interacts with the cell surface glycoprotein CD8A placenta–brain coexpressed, and *CSH1* (chorionic somatomammotropin hormone 1 or placental lactogen), *CSH2* (chorionic somatomammotropin hormone 2), *GH2* (growth hormone 2), and *VPREB1* (pre-B lymphocyte 1; also named *CD179A*, cluster of differentiation 179A) placental genes. *PTK7* (protein tyrosine kinase 7) expressed in the developing cortex, interacts with the peptide hormone CCK (cholecystokinin) from placenta and encodes a transmembrane receptor in the brain that controls a variety of developmental and physiological processes, including cell polarity, cell migration, invasion, and antagonize Wnt signaling (Peradziryi et al., 2011).

As mentioned above, placental 5-HT is an important regulator of fetal brain development. Our transcriptomic analysis suggests that other placental neuropeptides, as vasoactive intestinal peptide (VIP) and CCK could be related to the fetal brain development in humans. Moreover, according to comparative placental transcriptomic analysis (Hou et al., 2012), these neuropeptides are expressed in the human placenta but not in the mouse, cow or elephant placentas. Observations in mice showed that VIP has an influence over the brain and spinal cord development between E11 and E17. In the brain, VIP effects are specifically restricted to the cortex and tissue surrounding the ventricle. On the other hand, in the mouse the maternal levels of VIP are increased during E11, indicating a maternal VIP supply at early developmental stages from sources other than placenta (Hill et al., 1996; Hou et al., 2012). At difference, in humans has been found a placental source of VIP (Hou et al., 2012). Other studies have demonstrated that VIP can stimulate neurogenesis as well as differentiation and neurite outgrowth (Hill, 2007). Thus, maternal VIP from two different sources would be participating in the enlargement of the brain: a placental source observed in humans, and a non-placental source detected in mice.

Other gene interactions are elusive to be functionally interpreted, *GABBR1* [gamma-aminobutyric acid (GABA) B receptor 1] is expressed in the developing cortex and interacts with other GABA receptor in the placenta (*GABRG2*, GABA A receptor, gamma 2). *GABBR1* has been associated with anxiety (Le-Niculescu et al., 2011), autism (Fatemi et al., 2009), schizophrenia (Hegyi, 2013), and epilepsy (Peters et al., 1998), but there are not reports about brain development implications. *EDN* (endothelin 3) is an endothelium-derived vasoactive peptide expressed in the placenta, involved in a variety of biological functions and interacting with *EDNRA* (endothelin receptor type A), which is expressed in the developing cortex and upregulated after hypoxic preconditioning in the immature brain (Gustavsson et al., 2007). On the other hand, CCK has been identified in different brain areas through development. In mouse embryos, CCK expression first appears at E8.5–E9.5 in the neural crest cells and their precursors (Lay et al., 1999). In the embryonic rat brain CCK is expressed in the ventral tegmental area and in the primordium of the medial forebrain bundle from E15 onward. Cortical expression can be initially detected prenatally at E21 in rats (Cho et al., 1983). Thus, other CCK sources, as placenta in humans, would be relevant at earlier developmental stages. CCK functional significance in the intrauterine neurogenesis is unknown, however, CCK1 receptors have been associated to adult neurogenesis (Sui et al., 2013). Our transcriptome analysis revealed that VIP and CCK are associated with *CHGA* (chromogranin A or parathyroid secretory protein 1; Hocker et al., 1998), which encodes for the protein chromogranin A, a prohormone susceptible to be cleavage, generating catestatin, vasostatin and SgII-derived peptides like secretoneurin (Taupenot et al., 2003). These proteins are involved in the biogenesis of secretory granules,neurotransmitter accumulation, and in the control of neurosecretion (Montesinos et al., 2008). Chromogranin A is strongly associated with Alzheimer’s disease. It is localized in neuritic plaques where it can induce inflammation by activation of the microglia (Yasuhara et al., 1994; Hooper et al., 2009). On the other hand, a reduction in chromogranin A has been found in the layers III–VI of the prefrontal cortex (Brodmann area 9) of schizophrenic subjects, and associated to a lower number of presynaptic terminals and synaptic contacts, accompany by a decreased synaptic transmission (Iwazaki et al., 2004). These antecedents only demonstrate that chromogranin A has a possible functional role in the human brain. Studies about the role of this protein on brain development are needed to draw conclusions about the developmental cortical expression and its association with placental peptides. However, it is remarkable and intriguing that *CHGA* also is predicted to be interacting with the transcript of beta subunit of chorionic gonadotropin (CG). During pregnancy in some species,including human, CG is secreted by the trophoblast and maintains the progesterone secretion from the corpus luteum at the beginning of gestation. In humans, the production of CG declines around 6–8 weeks of pregnancy. Interestingly, receptors for luteinizing hormone (LH)/human chorionic gonadotropin (hCG) have been identified in multiple areas of the brain, including the cortex. Also, rat brain expresses these receptors and neurotropic effects of LH and hCG have been demonstrated in fetal rat brain (Lei and Rao, 2001). However, since there are other human placental functionally enriched modules and other species remain to be analyzed, this list can be easily extended in order to obtain a better understanding about the significance of this differential expression. We consider that these preliminary findings are complementary to the better characterized 5-HT signal originated in the placenta and interacting with the fetal forebrain at early developmental stages, the characterization of molecular interactions during development will open new opportunities to interpret evolutionary neurobiology and would reveal the causal relations in the pathogenesis of various cortical developmental disorders (Bonnin et al., 2011). With the exception of *PILRA* and *CECR1*, all human predicted transcripts are expressed in the developing cortex of the mouse. Because of this preserved pattern of cortical expression, species-specific interactions seem to be originated in the differentially activated transcripts in human placenta.

## CONCLUSION

The main idea proposed in this article is that the evolutionary expansion of the eutherian brain (specially in primates) would be associated with developmental long distance interactions through molecular signals displayed by the placenta. This hypothesis is complementary to those relied on the intrinsic control of neocortical development. In addition to the currently described 5-HT developmental interactions between the placenta and the cerebral cortical proliferative compartments, our transcriptomic analysis indicates new candidates for promoting neocortical expansion (e.g., *CSPG5*, *LGALS1*, *PTK7*). Thus, the active interaction between the placenta and the proliferative cortical compartments would amplify the number of neural progenitors as a strategy to increase the total number of neurons in the mature brain. If this proposal is correct, it not only allows to obtain a bigger brain, but also it would permit to reduce the length of the pregnancy required to generate bigger brains, supporting a developmental strategy to escape from fetal-maternal parental conflict. Interestingly, such interactions would conduce to fetal programming changes representing plausible adaptive advantages to environmental demands before and after birth, or maladaptation when there is a mismatch between the programming and the environment requirements (Hsiao and Patterson, 2012). For example, the known effect of maternal undernutrition over offspring metabolism and subsequent susceptibility to obesity later in life (Krechowec et al., 2006; Hsiao and Patterson, 2012). Several maternal insults, including maternal infection and maternal malnutrition, increase susceptibility to intrauterine growth restriction and all these factors are linked to schizophrenia, autism and cerebral palsy in the offspring (Brown and Susser, 2008; Atladóttir et al., 2010; Brown and Patterson, 2011; O’Callaghan et al., 2011). We are aware that detection of placental transcripts expression and their targets alone does not necessarily involves an effective functional interaction, therefore more studies are necessary to establish which mechanisms are implicated at the molecular level. However, according to our preliminary findings, it is remarkable that several pathways would be implicated in placenta–brain interactions and these could have a high impact in order to expand the current understanding of the evolutionary dynamics of neocortical expansion.

## Conflict of Interest Statement

The authors declare that the research was conducted in the absence of any commercial or financial relationships that could be construed as a potential conflict of interest.
